# Pharmacokinetic Study of Rectal Artesunate in Children with Severe Malaria in Africa

**DOI:** 10.1128/AAC.02223-20

**Published:** 2021-03-18

**Authors:** Caterina Fanello, Richard M. Hoglund, Sue J. Lee, Daddy Kayembe, Pauline Ndjowo, Charlie Kabedi, Benjamin B. Badjanga, Phettree Niamyim, Joel Tarning, Charles Woodrow, Melba Gomes, Nick P. Day, Nicholas J. White, Marie A. Onyamboko

**Affiliations:** a Centre for Tropical Medicine and Global Health, Nuffield Department of Medicine, University of Oxford, Oxford, United Kingdom; b Mahidol-Oxford Tropical Medicine Research Unit, Faculty of Tropical Medicine, Mahidol University, Bangkok, Thailand; c Kinshasa School of Public Health, University of Kinshasa, Kinshasa, Democratic Republic of Congo; d World Health Organization, Geneva, Switzerland

**Keywords:** antimalarial agents

## Abstract

When severe malaria is suspected in children, the WHO recommends pretreatment with a single rectal dose of artesunate before referral to an appropriate facility. This was an individually randomized, open-label, 2-arm, crossover clinical trial in 82 Congolese children with severe *falciparum* malaria to characterize the pharmacokinetics of rectal artesunate. At admission, children received a single dose of rectal artesunate (10 mg/kg of body weight) followed 12 h later by intravenous artesunate (2.4 mg/kg) or the reverse order. All children also received standard doses of intravenous quinine. Artesunate and dihydroartemisinin were measured at 11 fixed intervals, following 0- and 12-h drug administrations. Clinical, laboratory, and parasitological parameters were measured. After rectal artesunate, artesunate and dihydroartemisinin showed large interindividual variability (peak concentrations of dihydroartemisinin ranged from 5.63 to 8,090 nM). The majority of patients, however, reached previously suggested *in vivo* IC_50_ and IC_90_ values (98.7% and 92.5%, respectively) of combined concentrations of artesunate and dihydroartemisinin between 15 and 30 min after drug administration. The median (interquartile range [IQR]) time above IC_50_ and IC_90_ was 5.68 h (2.90 to 6.08) and 2.74 h (1.52 to 3.75), respectively. The absolute rectal bioavailability (IQR) was 25.6% (11.7 to 54.5) for artesunate and 19.8% (10.3 to 35.3) for dihydroartemisinin. The initial 12-h parasite reduction ratio was comparable between rectal and intravenous artesunate: median (IQR), 84.3% (50.0 to 95.4) versus 69.2% (45.7 to 93.6), respectively (*P* = 0.49). Despite large interindividual variability, rectal artesunate can initiate and sustain rapid parasiticidal activity in most children with severe *falciparum* malaria while they are transferred to a facility where parenteral artesunate is available. (This study has been registered at ClinicalTrials.gov under identifier NCT02492178.)

## Abstract (precis)

When severe malaria is suspected in children, the WHO recommends pretreatment with a single rectal dose of artesunate before referral to an appropriate facility. This was an individually randomized, open-label, 2-arm, crossover clinical trial in 83 Congolese children with severe *falciparum* malaria to characterize the pharmacokinetics of rectal artesunate.

## TEXT

Parenteral artesunate is the treatment of choice for severe *falciparum* malaria (1). Intravenous (i.v.) or intramuscular artesunate was associated with a substantial reduction in mortality compared with the previous first-line treatment, quinine (2, 3). Unfortunately, many children with severe malaria die before or just after reaching a facility capable of administering parenteral drugs. To address this need, a rectal formulation of artesunate has been developed that has been shown in very large community-based trials to reduce malaria mortality in children unable to tolerate oral medications reliably (4–10). These placebo-controlled trials were conducted in Ghana (*n* = 2,238, 6 to 72 months old), Tanzania (*n* = 3,802, 6 to 72 months old), and Bangladesh (*n* = 2,010, 6 to 72 months old; *n* = 4,018, older children and adults) (5). In young children in Africa and Asia, rectal artesunate was associated with a reduced risk (RR) of death compared to placebo (*n* = 8,050; RR = 0.74; 95% confidence interval [CI], 0.59 to 0.93). In older children and adults in Bangladesh, rectal artesunate was associated with a more than 2-fold increase in the risk of death compared to placebo (*n* = 4,018; RR = 2.21; 95% CI, 1.18 to 4.15; *P* = 0.01) (7). No satisfactory explanation was found for this paradoxical finding. One concern was the possibility of artesunate toxicity, as the absorption of rectal artesunate is erratic (11–13) and the dose given is around four times larger than the parenteral dose. Artesunate is rapidly metabolized, mainly by blood esterase and cytochrome P450 (CYP) 2A6, into its active metabolite, dihydroartemisinin (14). Dihydroartemisinin is metabolized via glucuronidation by uridine-diphosphate-glucanosyltransferase A1 (UGTA1), UGT1A9, and UGT2B7 into inactive metabolites, which are renally eliminated (15, 16). Artesunate and dihydroartemisinin have very short biological half-lives of less than 30 min and approximately 1 h, respectively, after both oral and parenteral administration of artesunate (17). To address concerns about a possible low efficacy and/or toxicity resulting from the erratic absorption of rectal artesunate that have negatively affected its deployment, we conducted a randomized crossover pharmacokinetic study of rectal artesunate versus intravenous artesunate in children with severe malaria in the Democratic Republic of the Congo at a time when parenteral quinine was still deployed as part of the first-line treatment of malaria.

## RESULTS

**FIG 1:**
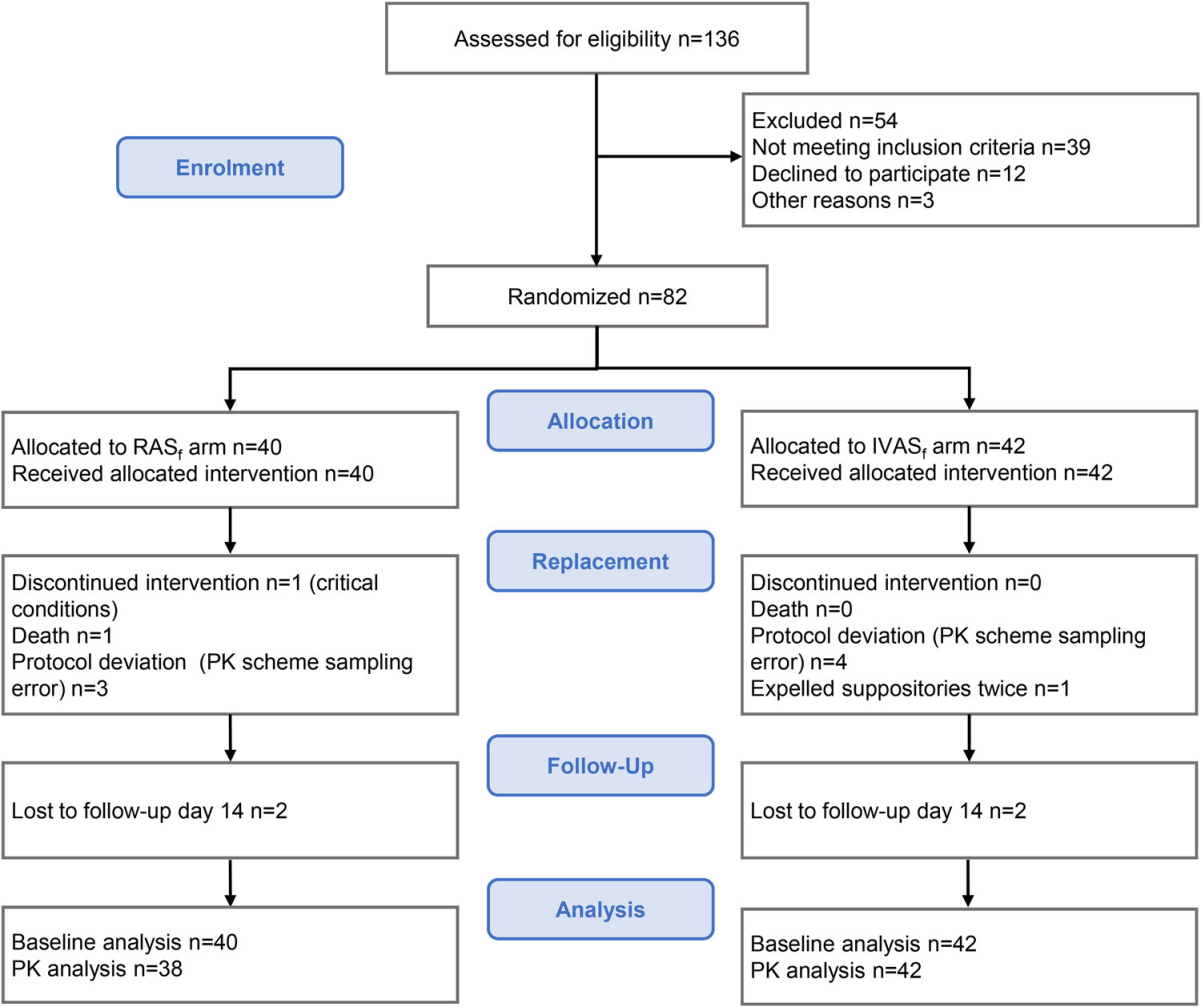
Flow chart CONSORT.

From 11 July to 6 October 2015, 136 patients with severe malaria were screened and 82 enrolled (Fig. 1). Ten patients were added to the original sample size (*n* = 72): in 7 cases a protocol deviation in the pharmacokinetic sampling scheme was reported, one patient expelled the study drug twice, one patient’s worsening conditions did not allow blood sampling, and one patient died 10 h after enrollment. The latter was retrospectively evaluated as having not met study inclusion criteria, as the child had received a full treatment of artemether-lumefantrine and an unidentified traditional medicine before coming to the hospital, but the information was disclosed to the staff only after enrollment. Available data from all 82 patients who were randomized and allocated to study treatments were included in this intention-to-treat analysis.

### Medical history.

According to medical history, children were brought to the center mainly because of fever (82/82, 100.0%; mean fever duration, 3.7 days; 95% CI, 3.4 to 4.1), severe prostration (64/82, 78.0%; mean duration, 1.6 days; 95% CI, 1.4 to 1.8), and convulsions (7/82, 8.5%). Other symptoms reported were gastrointestinal disorders (vomiting, abdominal pain, and nausea), anorexia, asthenia, and symptoms of an upper respiratory infection, with no significant differences between arms. Prior to admission, 3 children had received an artemisinin-based combination therapy (ACT), 19 oral quinine, 3 intramuscular artemether, and 1 intravenous (i.v.) quinine. The most common severity signs at screening were prostration (65/82, 79.3%), respiratory distress (64/82, 78.0%), coma (14/82, 17.1%), and severe anemia (25/82, 30.5%) (see Table S1 in the supplemental material). Comorbidities included acute renal failure (*n* = 1), gastritis and suspected gastric ulcer (*n* = 2), upper respiratory infection (*n* = 1), suspected sepsis (*n* = 2), and suspected meningitis (*n* = 3). At admission, the two treatment arms were well matched with no significant differences (Table 1 and Tables S2 to S4). Twenty-seven children were malnourished (27/79, 34.2%), 9 of whom were severely malnourished (malnourished was defined as a composite variable of wasted, stunted, and underweight). In 8 cases, 2 patients in the first arm who received rectal artesunate first (RAS_f_) and 6 in the first arm who received i.v. artesunate first (IVAS_f_), patients developed complications not present at admission or not reported by the caregiver. These included blackwater fever, convulsions, posturing, coma, deterioration of the coma score, severe anemia, and respiratory distress.

**TABLE 1:** Baseline data

Variable	RAS_f_	IVAS_f_
Evaluated (*n*)	40^*a*^	42^*a*^
Median age (IQR), yr	4.67 (2.8, 8.1)	4 (2.8, 8.8)
Median wt (IQR), kg	15.3 (12.0, 25.0)	14.3 (12.0, 24.5)
Median ht (IQR), cm	102 (89.0, 129.0); *n* = 39	98 (88.0, 131.0)
Median MUAC (IQR), cm	16.0 (14.5, 18.0)	15.2 (14.0, 17.0)
No. male (%)	21 (52.5)	20 (47.6)
No. with enlarged liver (%)	24 (60.0)	28 (66.7)
Median enlargement (IQR), cm	2.0 (0, 3.5)	3 (0, 4.0)
No. (%) with enlarged spleen	29 (72.50)	30 (71.43)
Median enlargement (IQR), cm	3.0 (0, 4)	3.0 (0, 4)
No. (%) with sickle cell trait	1/39 (2.6)	4/40 (10.0)
No. (%) with sickle cell disease	0/39	2/40 (5.0)
Mean HCT (SD), %	21.3 (7.6)	20.6 (7.1)
Mean Hb (SD), g/dl	7.1 (2.5)	6.9 (2.3)
GM parasite/μl (95% CI) screening	33,733 (15,031–75,702); *n* = 37	46,067 (19,484–108,920); *n* = 39
Median *Pf*HRP2 (range), ng/ml	1,674.1 (8.6–21,540.8); *n* = 39	1,442.8 (35.8–25,000.0); *n* = 42
Mean BP systolic (SD), mm Hg	90.6 (7.8); *n* = 38	92.6 (9.6); *n* = 41
Mean BP diastolic (SD), mm Hg	53.7 (7.4); *n* = 38	55 (6.7); *n* = 41
Median heart rate (IQR), bpm	146 (126, 163); *n* = 39	146.5 (123, 154)
Median respiratory rate (IQR), bpm	48 (40, 60)	44 (42, 52)
Mean temp (SD), °C	38.0 (1.1)	37.9 (1.1)
No. with blood transfusion (%)	26 (65.0)	27 (64.3)

a Unless indicated otherwise.

### Clinical and parasitological response to treatment. (i) Parasitemia.

Children with symptoms of severe malaria were enrolled if they had a positive malaria Ag Pf/Pan SD BIOLINE rapid diagnostic test at screening. The mean (geometric, 95% CI) peripheral blood parasitemia at admission was 33,733/μl (15,031 to 75,702) in the RAS_f_ arm and 40,067/μl (19,484 to 108,920) in the IVAS_f_ arm (*P* = 0.31). The mean (geometric, 95% CI) peripheral blood parasitemia at H0 was 40,111/μl (18,788 to 85,636) in the RAS_f_ arm and 40,658/μl (16,261 to 101,656) in the IVAS_f_ arm (*P* = 0.29). The median (range) plasma Plasmodium falciparum HRP2 (*Pf*HRP2) level was 1,674.1 ng/ml (8.6 to 21,540.8) in the RAS_f_ arm and 1,442.8 ng/ml (35.8 to 25,000.0) in the IVAS_f_ arm (*P* = 0.33) (Table 1). Retrospectively, 3 patients were negative by microscopy and 2 had only *falciparum* gametocytes; the plasma *Pf*HRP2 in these 5 cases ranged from 8.6 to 522.0 ng/ml. Two of these patients had received amodiaquine-artesunate and quinine tablets prior to admission and reported a history of fever of 5 and 7 days, respectively. Children who received a treatment before arriving at the center (documented or self-reported) had a lower parasitemia at admission (*n* = 23; 14,780/μl; 95% CI, 4,024 to 54,285) compared to those who did not (*n* = 53; 62,498/μl; 95% CI, 33,601 to 116,249, *P* = 0.06). The parasite reduction ratio (PRR) from hour 0 to 12 was comparable between arms, with a median (IQR) PRR of 84.3% (50.0% to 95.4%) in the RAS_f_ group and 69.2% (45.7% to 93.6%) in the IVAS_f_ group (*P* = 0.49). The estimated median (range) time for parasitemia to decrease by half was 2.2 h in the RAS_f_ arm (1.3 to 7.6) and 2.5 h in the IVAS_f_ arm (1.2 to 12.0), with no difference between arms (*P* = 0.64) (Tables 2 and 3). The limit of detection was 16 parasites/μl, and 1 case was excluded for insufficient data points.

**TABLE 2:** Summary of parasite clearance time by study arm

Parameter	RAS_f_	IVAS_f_	*P* value
No. of individual profiles analyzed	35	40	
Slope half-life, h			0.64
Median	2.2	2.5	
Range	1.3–7.6	1.2–12.0	
GM (95% CI)	2.3 (2.0; 2.6)	2.43 (2.1; 2.8)	
*t*-lag, h			0.81
Median	0	0	
Range	0–12	0–24	
IQR	0–6	0–6	
GM (95% CI)	6.9 (5.9–8.1); *n* = 5	8.3 (6.2, 11.0); *n* = 15	
Median pc50 (range), h	7.1 (0.3, 15.1)	6.8 (0.4, 24.4)	0.28
Median pc90 (range), h	11.8 (4.1, 25.7)	13.9 (3.5, 41.8)	0.16
Median pc95 (range), h	14.0 (5.7, 33.3)	16.8 (4.8, 53.8)	0.13
Median pc99 (range), h	18.8 (9.1, 50.8)	22.1 (7.8, 81.7)	0.14

**TABLE 3:** Hematology at 0 and 12 h by arm

Parameter	RAS_f_	IVAS_f_	*P* value
Mean (SD) hemoglobin			
At 0 h	7.1 (2.6); *n* = 39	6.88 (2.3); *n* = 42	
At 12 h	9.1 (1.6); *n* = 38	8.7 (2.2); *n* = 42	
Median (IQR) within individual differences (from H0 to H12)	−2.5 (−4.3, 0.7); *n* = 80	−2.2 (−3.9, 0.6); *n* = 80	0.75
Geometric mean (95% CI) parasitemia			
At 0 h	40,111 (18,788, 85,636); *n* = 36	40,658 (16,261, 101,656); *n* = 40	
At 12 h	5,420 (1,853, 15,851); *n* = 34	8,518 (2,721, 26,667); *n* = 38	
Median (IQR) within individual differences (from H0 to H12)	6.3 (2.0, 18.1); *n* = 72	3.0 (1.8, 12.0); *n* = 72	0.37

### (ii) Hematology.

The mean (standard deviation [SD]) hemoglobin (Hb) at H0 was 7.1 (2.5) g/dl in the RAS_f_ arm and 6.9 (2.3) g/dl in the IVAS_f_ arm. Children with the sickle cell trait (*n* = 5) had a mean (SD) Hb of 6.5 (3.1) g/dl, while the two children with sickle cell disease had 3.7 and 3.9 g/dl at admission. Fifty-three children received a blood transfusion, 26 (65.0%) in the RAS_f_ arm and 27 (64.3%) in the IVAS_f_ arm (*P* = 0.95). From admission to 12 h (before the crossover), the median (IQR) difference in Hb was −2.5 (−4.3, 0.7) in the RAS_f_ arm and −2.2 (−3.9, 0.6) in the IVAS_f_ arm (*P* = 0.75) (Table 3). By day 7, the mean Hb (SD) was 9.6 (1.4) g/dl in the RAS_f_ arm (*n* = 38) and 9.3 (1.6) g/dl in the IVAS_f_ arm (*n* = 41, *P* = 0.46 adjusted for baseline Hb), and by day 14 the values were 10.6 (1.0) g/dl in the RAS_f_ arm (*n* = 36) and 10.2 (1.5) g/dl in the IVAS_f_ arm (*n* = 40, *P* = 0.21, adjusted for baseline Hb).

### Follow-up visits and neurological assessment.

The median time of hospitalization was 3 days (range, 3 to 14 days). There were no significant differences between arms in the recovery time (median time from admission to sit unsupported [*P* = 0.74], speak [*P* = 0.41], localize painful stimuli [*P* = 0.58], and eat/breastfeed [*P* = 0.95]). Only one patient (randomized to the IVAS_f_ arm; received 11.8 mg/kg of body weight rectal artesunate at 12 h) developed neurological sequelae (unable to walk); the patient was admitted with a Glasgow coma score of 9/15, 9,546 parasites/μl, Hb of 6.7 g/dl, and temperature of 38.0°C, developed convulsions and posturing after admission, and was in the hospital for 4 days. The sequelae completely resolved by day 14.

### Adverse events.

Forty-nine patients (59.8%) reported one or more adverse events of mild or moderate intensity, without differences between arms. Most patients had fluctuations in the electrolytes (*n* = 31) or white blood cell (WBC) and platelet counts (*n* = 7) above or below the normal range. One child developed pruritus after i.v. artesunate administration; this was classified as possibly related. The remaining cases were all classified as likely unrelated or unrelated to drug administration (Table 4).

**TABLE 4:** List of adverse events

Adverse event	RAS_f_	IVAS_f_	Total
Electrolyte changes	15	16	31
WBC/platelet changes	2	5	7
Expelled artesunate suppository^*a*^	5	5	10
Pruritus/cutaneous rash^*a*^^,^^*b*^	0	1	1
Urticaria^*c*^	0	1	1
Viral/bacterial infection suspected	2	5	7
Intestinal parasite infection suspected	1	1	2
Vomit	0	1	1
Epistaxis	0	1	1
Swollen face/acute renal failure	0	1	1
Hypersialosis/acute renal failure	0	1	1
Conjunctivitis (day 14)	1	0	1
Gastroenteritis (day 14)	1	0	1

a Classified as possibly related.

b Developed after i.v. artesunate administration.

c Developed 8 min after blood transfusion started.

### Rectal artesunate administration.

In the RAS_f_ arm, 5 patients expelled the suppositories within 60 min (range, 2 to 29 min), and a new dose was administered. In the IVAS_f_ arm, 5 patients expelled the suppositories within 60 min (range, 10 to 15 min), and a new dose was administered. One patient also expelled the second dose. These 10 events were classified as possibly related to rectal artesunate administration. Children in the first weight group (6.0 to 12.9 kg) received (median, IQR) 9.1 (8.3 to 10.0) mg/kg rectal artesunate, 12.5 (10.7 to 14.3) mg/kg in the second group (13.0 to 23.9 kg), and 10.7 (9.8 to 11.8) mg/kg in the third group (24.0 to 34.0 kg) (Table S5).

### Pharmacokinetic properties of rectal artesunate.

**FIG 2:**
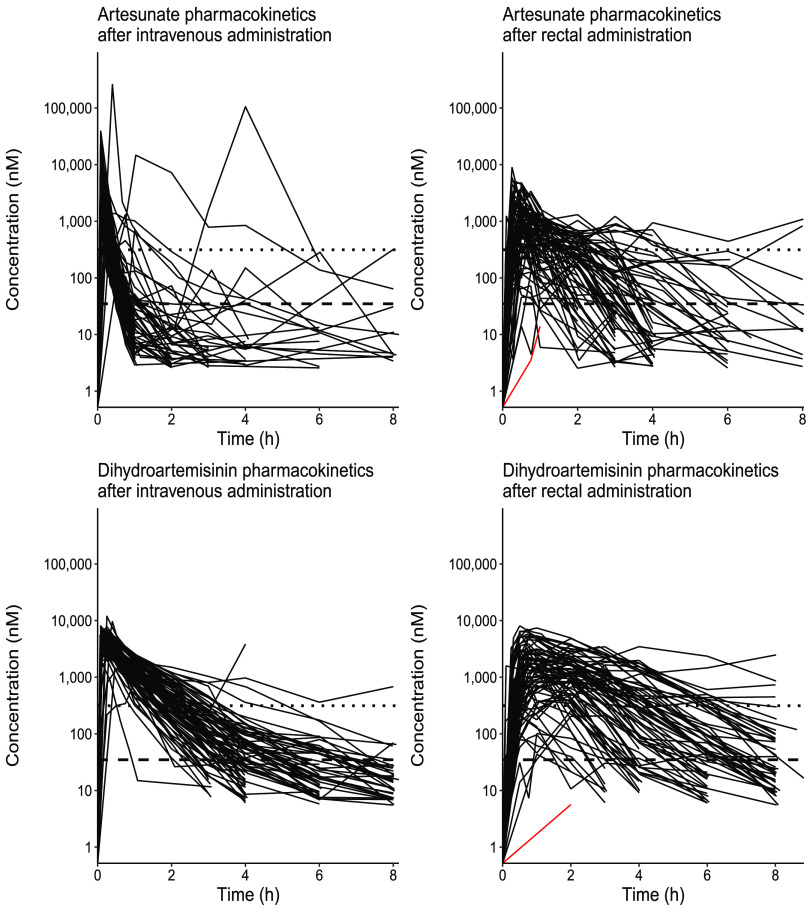
Observed individual artesunate concentration-time profiles. Artesunate and dihydroartemisinin were administered intravenously or rectally. The dashed horizontal line represents a putative IC_50_ value of 34.9 nM, and the dotted horizontal line represents a putative IC_90_ value of 314 nM. The red line represents the patient who did not reach the IC_50_ value after rectal administration.

**FIG 3:**
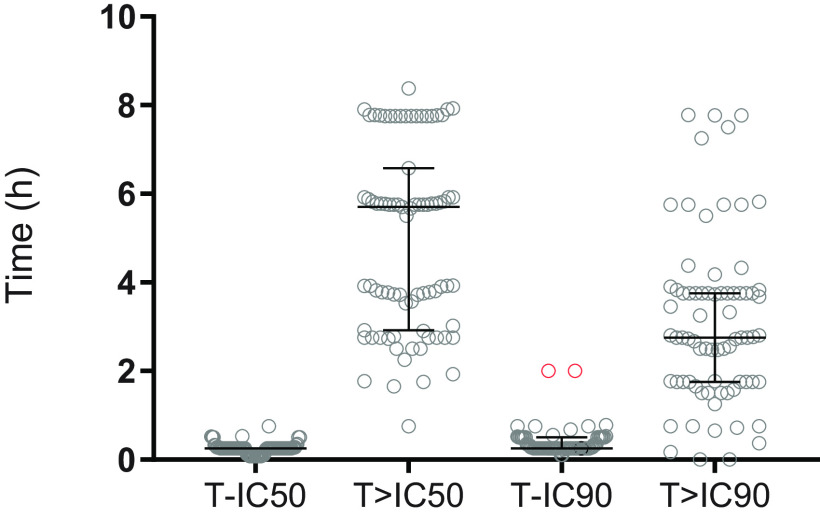
Graphical representation of time to putative IC_50_ (T-IC_50_), time above IC_50_ (T>IC_50_), time to putative IC_90_ (T-IC_90_), and time above IC_90_ (T>IC_90_) after rectal artesunate administration. Concentrations were measured as the sum of molar artesunate and dihydroartemisinin concentrations. Markers represent individual values and lines represent the median value and its interquartile range. The graphic shows only data for patients who reached the cutoff (IC_50_ or IC_90_). One patient did not reach IC_50_. The red dots indicate the 2 cases with a longer time to reach IC_90_.

Data from 80 patients (two patients with data from only 1 time point were excluded) were used to evaluate the pharmacokinetic properties of artesunate and dihydroartemisinin after intravenous and rectal administration. After rectal administration, both artesunate and dihydroartemisinin reached peak plasma concentrations relatively fast, resulting in a median (IQR) time to reach maximum concentration (*t*_max_) of 0.5 h (0.25 to 0.75) for artesunate and 1.0 h (0.75 to 2.00) for dihydroartemisinin. However, individual concentration-time profiles of both artesunate and dihydroartemisinin showed large interindividual variability in both arms, especially after rectal administration (Fig. 2). The absolute peak concentrations of dihydroartemisinin varied between 5.63 nM and 8,090 nM after rectal administration (i.e., 1,000-fold variation). Almost all patients (79/80, 98.7%) reached the putative 50% inhibitory concentration (IC_50_) value of 34.9 nM, and most patients (74/80, 92.5%) reached the IC_90_ value of 314 nM at a median (IQR) time of 0.25 h (0.25 to 0.25) and 0.25 h (0.25 to 0.50), respectively (Fig. 3). Time above the putative IC_50_ varied between patients, with a median of 5.68 h (2.90 to 6.08) above the IC_50_ value and 2.74 h (1.52 to 3.75) above the IC_90_ value (Fig. 3). Two patients had a zero duration above the IC_90_, since only one observation was above the cutoff. The median (IQR) rectal bioavailability was estimated to be 25.6% (11.7 to 54.5) for artesunate and 19.8% (10.3 to 35.3) for dihydroartemisinin, emphasizing the large interindividual pharmacokinetic variability. Similar results were obtained if all patients that expelled the suppositories were excluded from the pharmacokinetic analysis. A detailed description of patients who did not reach IC_50_ (*n* = 1) or reached IC_90_ later than others (*n* = 2) is presented in the supplemental material.

Compared to intravenous artesunate, rectal artesunate had a lower (median [IQR]) maximum concentration (*C*_max_, in nM) (442 [213 to 813] versus 2,560 [1,450 to 5,200]), later time to reach the maximum concentration (*t*_max_, in h) (0.500 [0.250 to 0.750] versus 0.083 [0.083 to 0.100]), and lower total drug exposure (AUC*_t_*, area under the concentration-time curve until the last measurable concentration, in h·nM/μmol) (2.57 [1.68 to 4.57] versus 10.20 [5.92 to 14.00]). The terminal elimination half-life (*t*_1/2_, in h) was comparable between intravenous and rectal artesunate (0.525 [0.325 to 0.770] versus 0.571 [0.299 to 1.08]) (Table 5). The exposure and bioavailability of artesunate and dihydroartemisinin were comparable between patients who received rectal artesunate at time zero and those who received it at 12 h. Similarly, exposure was comparable after i.v. artesunate regardless of the time of administration. Children received a median (IQR) of 10.5 mg/kg (9.1 to 12.0) rectal artesunate, corresponding well with the intended target dose (Table S5). Fourteen children (17.0%) received less than 9.0 mg/kg (median [IQR], 8.3 mg/kg [8.0 to 8.3]), and 13 children (15.9%) received more than 13.0 mg/kg (14.3 mg/kg [13.3 to 15.4]). There were no significant differences in the pharmacological parameters (bioavailability, *P* = 0.37 for artesunate and *P* = 0.57 for dihydroartemisinin) or pharmacodynamic parameters (parasite clearance time, *P* = 0.90) between children who received a higher (≥13.0 mg/kg, *n* = 13) or lower (<9.0 mg/kg, *n* = 14) dose compared to the target dose (9.0 to 12.9, *n* = 55). No significant differences were observed between patients who received a blood transfusion after admission and those who did not within each treatment arm. After rectal artesunate administration, nourished (*n* = 54), malnourished (*n* = 17), and severely malnourished (*n* = 9) children had significantly different median (IQR) exposure (AUC*_t_*/dose) to artesunate (2.28 [1.12 to 3.52] h·nM/μmol, 2.32 [1.71 to 5.19] h·nM/μmol, and 4.99 [4.36 to 6.45] h·nM/μmol, respectively; *P* = 0.04) and dihydroartemisinin (5.87 [3.38 to 11.1] h·nM/μmol, 7.24 [4.93 to 14.4] h·nM/μmol, and 16.5 [7.96 to 25.8] h·nM/μmol, respectively; *P* = 0.02). In contrast, bioavailability was comparable between these three groups (*P* > 0.05).

**TABLE 5:** Pharmacometric parameters of artesunate and dihydroartemisinin after intravenous and rectal administration of artesunate in children with severe malaria

Parameter^*e*^	Value(s) [median (IQR)] by route of administration	
	Intravenous	Rectal
Analyzed, *n*	80	80
Artesunate		
*t*_max_ (h)	0.083 (0.083–0.100)	0.500 (0.250–0.750)
*C*_max_ (nM)	6,660 (3,770–13,500)	442 (213–813)
*C*_max_/*D* (nM/μmol)	71.0 (40.6–99.1)	2.08 (1.05–4.26)
*t*_last_ (h)	3.00 (2.00–6.00)	6.00 (4.00–8.00)
AUC*_t_*/*D* (h·nM/μmol)	10.2 (5.92–14.0)	2.57 (1.68–4.57)
*t*_1/2_ (h)	0.571^*a*^ (0.299–1.08)	0.525^*b*^ (0.325–0.770)
*F* (%)		25.6 (11.7–54.5)
Dihydroartemisinin		
*t*_max_ (h)	0.250 (0.083–0.250)	1.00 (0.750–2.00)
*C*_max_ (nM)	5,100 (3,850–5,860)	1,800 (822–3,100)
*C*_max_/*D* (nM/μmol)	40.8 (28.6–74.3)	3.34 (1.88–6.44)
*t*_last_ (h)	8.00 (6.00–8.00)	8.00 (7.89–8.00)
AUC*_t_*/*D* (h·nM/μmol)	37.4 (24.3–61.2)	6.97 (3.94–14.5)
*t*_1/2_ (h)	0.882^*c*^ (0.686–1.19)	0.865^*d*^ (0.681–1.18)
*F* (%)		19.8 (10.3–35.3)

a One individual was excluded from the analysis due to a lack of data in the elimination phase.

b Six individuals were excluded from the analysis due to a lack of data in the elimination phase.

c One individuals were excluded from the analysis due to a lack of data in the elimination phase.

d Seven individuals were excluded from the analysis due to a lack of data in the elimination phase.

e *D*, dose.

## DISCUSSION

This pharmacokinetic study in children with severe *falciparum* malaria showed that artesunate is rapidly absorbed by most patients after rectal administration: 98.7% of children reached the IC_50_ within a median (IQR) time of 0.25 h (0.25 to 0.50), and 92.5% of them reached the IC_90_ within 0.25 h (0.25 to 0.50). The median time spent above the IC_50_ was more than 5 h, indicating that the rectal formulation will start and continue its parasiticidal activity during transportation to a medical facility. Rectal artesunate suppositories could reduce parasitemia rapidly, as shown by the similar rates of parasitemia reduction after treatment with either rectal or parenteral artesunate. Therefore, any differences in pharmacokinetic parameters between arms and the variability observed did not translate into a worse pharmacodynamic profile, confirming previous results in studies of patients with severe and moderately severe *falciparum* malaria (6, 11). Artemisinin derivatives clear parasitemia more rapidly than other drugs and, by acting on ring stages, prevent parasites from maturing and sequestering. In contrast, quinine acts only in a limited manner on ring stages (18), and the initial parasitemia reduction observed, although dependent upon the mean age of development of circulating parasites, is typically slower than that with artemisinin derivatives (19). Therefore, we assume that the administration of quinine in our study affected only a negligent proportion of the peripheral parasitemia, and the fast parasite reduction observed was mainly the result of the absorbed artesunate. These results support giving prereferral artesunate to all children suspected of having malaria who cannot reliably take oral medications, including children who might have otherwise uncomplicated malaria with repeated vomiting to profoundly ill unconscious children. The quick absorption of artesunate after rectal administration is encouraging, but the formulation also exhibits a high variability in exposure due to the very high variability in absorption. However, as rectal artesunate is not a replacement for intravenous treatment but instead an early start of the treatment while being transported to the hospital, the total exposure is not the most important pharmacokinetic parameter. Instead, it is important to absorb enough of the drug to reach effective concentrations rapidly. Most children received appropriate doses of rectal artesunate, according to the target dose of 10 mg/kg, and no significant differences in bioavailability or parasitemia reduction were observed in the groups that received lower or higher doses, allaying any potential concerns related to a reduced efficacy or toxicity. Although the study was not designed or sufficiently powered to detect a difference in the rate of adverse events between treatments, the number was low and comparable between arms. Malnutrition is frequent in areas where malaria is endemic, and it has been associated with an increased risk of reduced antimalarial drug exposure (20). In this study, malnourished and severely malnourished children had a slightly higher drug exposure than the other children. The severely malnourished group was small in the present study, and a larger study would be needed to investigate this result further. In line with 2010 WHO recommendations (21), rectal artesunate was included in the National Guidelines of the DRC in 2012, although its deployment on a large scale has since been delayed (22). The results of this study support country-wide deployment of this intervention in the Democratic Republic of Congo.

### Conclusions.

Clearly, parenteral artesunate is preferable to rectal administration, but this is not an option in many villages and rural health centers in resource-limited areas. Despite large interindividual variability, rectal artesunate can initiate and sustain rapid parasiticidal activity in most children with severe *falciparum* malaria while they are transferred to a facility where parenteral artesunate is available.

## MATERIALS AND METHODS

### Study site.

This trial was conducted by the Kinshasa School of Public Health–University of Oxford Medical Research Unit (KIMORU) team at Kingasani Hospital, Kinshasa, the Democratic Republic of Congo. Malaria transmission in the area is high and perennial.

### Trial design.

This was an individually randomized, open-labeled, 2-arm, crossover clinical trial in children admitted to the hospital with severe malaria (23). A weight group-stratified 1:1 randomization design was used, with three blocks for each arm according to body weight (6.0 to 12.9 kg, 13.0 to 23.9 kg, and 24.0 to 34.0 kg), to have the same number of patients administered 1, 2, or 3 suppositories. A computer-generated randomization list was prepared by a study statistician. Treatment allocation was concealed in sequential opaque envelopes prepared by an independent person. The intervention was assigned by the study nurse after the doctor confirmed eligibility and the caregiver had signed the informed consent. When the envelope was opened and signed, the patient was considered enrolled.

### Eligibility criteria for participants.

Children were included in the study if they fulfilled the WHO criteria for severe *falciparum* malaria (23), had a weight of ≥6.0 kg and ≤34.0 kg, had a positive malaria Ag Pf/Pan SD BIOLINE rapid diagnostic test, and their parent or caretaker gave fully informed consent. Children were not included if they had acute diarrhea (defined as >3 liquid stools in the previous 24 h), visible anorectal malformations or a disease of the rectum, known hypersensitivity to quinine or artesunate, a documented history of an effective dose of parenteral antimalarial in the preceding 24 h, a single dose of rectal artesunate in the previous 12 h, a dose of an ACT in the previous 6 h, a comorbidity that could have interfered with the study or put the patient at risk, or participation in another clinical trial or earlier in the same clinical trial.

### Interventions.

Children were randomized to receive either 1 dose of rectal artesunate (approximating as closely as possible to 10 mg/kg) on admission (time zero) followed 12 h later by intravenous artesunate (2.4 mg/kg) (RAS_f_ arm) or the reverse order (IVAS_f_ arm). Children were observed for 1 h, and if the suppository was expelled within 60 min, a single attempt was made to readminister a second dose (the second dose was equal to the number of suppositories expelled). As the absorption of rectal artesunate is known to be erratic, all children were given intravenous quinine (20 mg salt/kg loading dose at presentation followed by 10 mg/kg every 8 h) by rate-controlled infusion for a total of three doses (1). Quinine and artesunate can be administered concomitantly without risk (24). The quinine infusion was started immediately after the study drug. If a blood transfusion was needed at admission, quinine was started after the blood transfusion was terminated, although the first dose of the study drug was not delayed. After 24 h, all patients continued antimalarial therapy with parenteral artesunate, followed by a full standard course of artemether-lumefantrine as soon they could take oral medication. If the child was discharged before the oral treatment was terminated, the remaining doses were given to the caregiver for home administration.

### Study drugs.

Intravenous artesunate (Guilin Pharmaceuticals, China), intravenous quinine (Rotex, Germany), and Coartem (Novartis) were purchased from Medical Expert Group, Gorinchem, The Netherlands. Rectal artesunate, in suppositories of 100 mg each, was produced by Catalent, Germany Eberbach, GmbH, packed by Scanpharm, Copenhagen, Denmark, and provided by the World Health Organization for this study.

### Outcomes.

The primary objective of the study was to assess the pharmacokinetics of rectal artesunate in pediatric patients with severe P. falciparum malaria. The secondary objective was characterization of the clinical and parasitological responses to rectal artesunate compared to intravenous artesunate. A randomized sequence crossover design was employed to characterize the bioavailability of rectal artesunate and to characterize the individual absorption profiles of both artesunate and dihydroartemisinin. From a therapeutic perspective, rectal artesunate aims to achieve minimum parasiticidal concentrations (MPC) as soon as possible. Both artesunate and dihydroartemisinin exhibit parasiticidal effects; therefore, the sum of the molar artesunate and dihydroartemisinin concentrations were evaluated, and the time to reach, time above, and the proportion of patients that reached a putative IC_50_ and IC_90_ (i.e., 34.9 nM and 314 nM, respectively) were considered the primary end points, with conventional pharmacokinetic measures as secondary end points (e.g., bioavailability). The IC_50_ value was taken from the estimated EC_50_ (concentration in the dihydroartemisinin effect compartment) (25), and the IC_90_ was calculated from the IC_50_.

### Investigations.

Malaria at screening was confirmed by Malaria Ag Pf/Pan SD BIOLINE rapid diagnostic test. A malaria blood film was prepared at admission and 0 (predose), 6, and 12 h and every 12 h thereafter until 2 consecutive blood films were negative. Parasites were identified and counted by standard light microscopy. Hemoglobin (Hb) and hematocrit (Hct) were assessed at the same time points as the blood films using HemoCue Hb301+ (Angelholm, Sweden) and Hawksley Haematospin 1400 (Hawksley & Sons, Ltd., UK). Total and differential white blood cell (WBC) counts were measured using QBC Star at 0, 24, and 72 h. Biochemistry tests were performed at 0 and 24 h by i-STAT using the CHEM8 cartridge for electrolytes and the CG4 cartridge for blood gases. Hemoglobin S trait was detected by electrophoresis using the SEBIA Hydragel hemoglobin K20 kit. The quantification of plasma *Pf*HRP2 by enzyme-linked immunosorbent assay (ELISA; Celisa; Cellabs, Sydney, Australia) was performed at the Mahidol Oxford Tropical Medicine Research Unit (MORU) laboratories, Bangkok, Thailand. Laboratory technicians were blinded to study treatment allocation.

### Pharmacokinetic blood sampling.

Eleven blood samples were taken at fixed intervals: pretreatment, 5, 15, 30, and 45 min and 1, 2, 3, 4, 6, and 8 h after the administration of the first dose of study drug. The sampling scheme was repeated after 12 h following the administration of the second dose of the study drug. Blood was sampled through an indwelling cannula in the arm opposite that used for intravenous drug administration; 1 ml of blood was collected into prechilled fluoride oxalate tubes for artesunate and dihydroartemisinin quantification. Samples were centrifuged at 4°C and 2,000 × *g* for 7 min. Plasma samples were stored at −80°C until they were shipped to the MORU Department of Clinical Pharmacology, Bangkok, Thailand, for drug quantification. Artesunate and dihydroartemisinin were quantified using liquid chromatography-tandem mass spectrometry (26). The coefficient of variation of the assay was less than 7% at each level of quality control samples, and the lower limit of quantification (LLOQ) was set to 1.19 ng/ml and 1.96 ng/ml for artesunate and dihydroartemisinin, respectively.

### Patient management.

Patients were managed according to WHO guidelines for the management of severe malaria (1). Fever was treated with parenteral paracetamol at 20 mg/kg. Hypoglycemia (blood glucose of <3 mmol/liter) was treated with an i.v. bolus of 5 ml/kg 10% dextrose. A blood transfusion (20 ml/kg) was given to children with hemoglobin concentrations of <5 g/dl. A fluid bolus was given to children with signs of shock. Convulsions were treated with i.v. diazepam. All children were given ceftriaxone intravenously (75 mg/kg at time zero and after 12 h).

### Assessment at follow-up visits.

Patients were hospitalized for at least 4 days, longer if they were still unwell, and discharged after at least the first dose of the oral follow-on treatment was administered. Parents/guardians were asked to bring the child back to the clinic at day 7 (if they were discharged earlier) and 14 for clinical examination, neurological exam, and laboratory tests.

### Ethics.

The study was approved by the Ethical Committee of the Kinshasa School of Public Health, the Ministry of Public Health of DRC, and the Oxford University Tropical Research Ethics Committee (OXTREC). The study documents were originally designed in English. The protocol was translated into French and the Patient Information Sheet and Informed Consent into French and Lingala by a certified translator. Safety reporting was performed according to the ICH Harmonized Tripartite Guideline for Good Clinical Practice (1996).

### Sample size.

This was considered a bioequivalence study, comparing a new formulation (rectal) to a reference (intravenous). Previous data in uncomplicated and moderately severe malaria in pediatric patients showed that rectal artesunate is characterized by a large inter- and intraindividual variability (11–13). Assuming a within-subject coefficient of variation (CV) of 40% (27, 28), a sample size of 72 patients was estimated to be sufficient to assess the bioequivalence of the two drugs, with 90% power and 95% confidence, including 10% loss to follow-up. This sample size was calculated using the formula by Julious for crossover studies (29).

### Statistical analysis.

Medical histories of patients were described using frequencies (percent) for each study arm. Clinical and parasitological responses to treatment were reported using geometric means (GM) with 95% confidence intervals (CI), median (interquartile range [IQR] or range), or mean (SD) and compared between treatment arms using the Kruskal-Wallis test or Student's *t* test. Hb comparisons after baseline were adjusted for baseline values of Hb. Parasite clearance half-life (PC_1/2_), lag time (*t*-lag), and the time to clear 50, 90, 95, and 99% of parasites was calculated using the Parasite Clearance Estimator, developed by WWARN (30), which was modified to allow for a lower threshold of parasitemia at time zero. The parasite reduction ratio was calculated as the difference at 12 h from baseline divided by the baseline parasite count. Pharmacokinetic parameters were compared between treatment arms as well as between children who received higher (≥13.0 mg/kg) or lower (<9.0 mg/kg) doses than the target dose (9.0 to 12.9) between nourished, malnourished, and severely malnourished children and between patients who did or did not receive a blood transfusion. The subgroup of children who did not expel their suppositories was also assessed. Statistical analyses were performed using STATA IC 14.0 (STATA Corporation, College Station, TX) and GraphPad Prism Software (San Diego, CA).

### Pharmacokinetic analysis.

Artesunate and dihydroartemisinin concentration-time profiles were analyzed on an individual level using a standard noncompartmental approach in Phoenix 64 (Certara USA, Inc., Princeton, NJ). All concentrations at time zero were set to zero. The concentration at the time point when the concentration-time profile went permanently below the LLOQ for the first time was set to half the LLOQ. Artesunate data after i.v. administration were analyzed assuming an infusion using the true injection times for patients, while the rectal administration as well as the dihydroartemisinin data after i.v. and rectal administration were handled as extravascular administration. The observed concentration-time profiles were used to derive the maximum concentration (*C*_max_), the time to maximum concentration (*t*_max_), and the time to the last measured concentration (*t*_last_). Total drug exposure (area under the concentration-time curve; AUC*_t_*) was calculated using the observed data from drug administration to the last time point. The calculations of AUC were based on the trapezoid method, using the linear method before *C*_max_ and the logarithmic method after *C*_max_. Terminal elimination half-life (*t*_1/2_) was based on the best fit of the terminal portion of the elimination phase. Absolute rectal bioavailability (*F*) was estimated based on individual drug exposures after rectal and intravenous administration, according to the following equation:

$$F=\frac{{\text{AUC}}_{t,\text{rectal}}}{{\text{AUC}}_{t\text{,i.v.}}}\times \frac{{\text{Dose}}_{\text{i.v.}}}{{\text{Dose}}_{\text{rectal}}}$$

The time to reach a putative IC_50_ and IC_90_ value (25), the time spent above these values, and the proportion of patients who reached this value were derived directly from the observed concentration-time profiles. Pharmacokinetic parameters from each mode of administration were compared using a Mann-Whitney U test to determine the effect of drug administration time (0 or 12 h) and of blood transfusion and with Kruskal-Wallis test to compare severely malnourished, malnourished, and nourished children.

### Data availability.

The data that support the findings are available from the authors upon reasonable request and with permission of the University of Oxford and the Kinshasa School of Public Health.

## Supplementary Material

Supplemental file 1
