# Supplementary material for: Pharmacokinetic Study of Rectal Artesunate in Children with Severe Malaria in Africa
Source: Antimicrob Agents Chemother. 2021 Mar 18;65(4):e02223-20. doi: 10.1128/AAC.02223-20 (PMC8097454; doi:10.1128/AAC.02223-20)
Supplement: Supplemental file 1 [file AAC.02223-20-s0001.pdf]

Supplementary materials

Figure S1. Study design

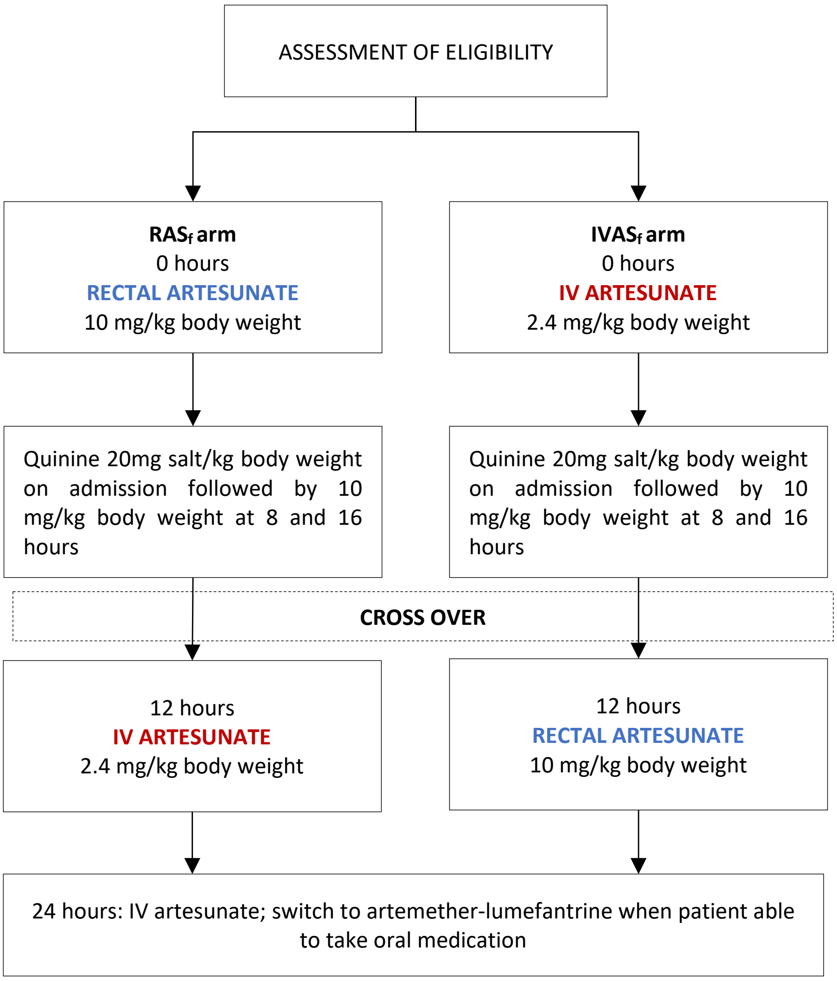

Table S1. Severity signs at screening

Malaria severity signs, N (%)	RAS_f	$IVAS_f$
Evaluated [a]	40	42
Prostration	32 (80.0)	33 (78.6)
Respiratory distress	31 (77.5)	33 (78.6)
Severe anaemia	13 (32.5)	12 (28.6)
Coma	6 (15.0)	8 (19.1)
Convulsions	3 (7.5)	2 (4.76)
Decompensated shock	4 (10.0)	1/41 (2.4)
Hyperlactatemia (>5mm/L)	11/35 (31.4)	8/32 (25.0)
**Acidosis (plasma bicarbonate < 15 mmol/L)**	3/38 (7.9)	3/41 (7.3)
Hypoglycaemia	3 (7.5)	1 (2.4)
Jaundice	3 (7.5)	2 (4.8)
Hyperparasitaemia (>500,000/µL)	2 (5.1)	5 (11.9)
Anuria/oliguria	1 (2.5)	1 (2.4)
Renal impairment (serum creatinine >265 mol/L)	1 (2.5)	0

[a] Unless indicated otherwise

Table S2. Physical examination at admission

No. patients with abnormality (%)	RAS_f	$IVAS_f$
Evaluated [a]	40	42
Eyes (conjunctiva pallor, convergent strabismus, jaundice)	24 (60.0)	27 (60.5)
ENT (nasal discharge)	2 (5.0)	1 (2.4)
Cardiovascular system (tachycardia)	32 (80.0)	35 (83.3)
Respiratory system (tachypnoea)	32 (80.0)	38 (90.5)
GI (epigastric pain)	1/38 (2.6)	1 (2.4)
Skin (skin pallor, dehydration)	23 (57.5)	23 (54.8)
Joints (joint pain)	1 (2.5)	0
Temperature gradient leg	13 (32.5)	8 (19.1)

[a] Unless indicated otherwise

Table S3. Neurological examination at admission

Neurological signs, N (%)	RAS_f	$IVAS_f$
Evaluated [a]	39	41
Posturing (%)	0	1 (2.4)
Neck stiffness (%)	0/37	0
Convulsions (%)	1 (2.6)	0
Bulging fontanelle (%)	0/27	0/25
No. with GCS score	33	34
Median score (min, max)	15 (8, 15)	15 (8, 15)
No. with BCS score	6	7
Median score (min, max)	5 (3, 5)	5 (1, 5)

[a] Unless indicated otherwise

Table S4. Respiratory rate by age group at admission

Age group	N	Mean	Range	SD
0 to 1 year	1	60	-	-
1 to 3 years	28	56	26-82	11.4
3 to 6 years	24	48	32-80	11.7
> 6 years	29	42	28-60	7.7

Table S5. Total mg. of rectal artesunate by weight group

Body weight (kg)	N	mg. AS	median (IQR) mg. AS /kg/body weight
6.0-12.9	30	100 mg	9.1 (8.3-10.0)
13.0-23.9	28	200 mg	12.5 (10.7 -14.3)
24.0-34.0	24	300 mg	10.7 (9.8-11.8)
**Overall**	82		10.5 (9.1-12.0)

Descriptions of the cases not reaching IC_{50} or reaching IC_{90} later than others

Case 1 (did not reach IC_{50}) Received 10.0 mg/kg of rectal artesunate at 12 hr. Screening parasitaemia 176/μL, *pf*HRP2 4,767 ng/mL, Hb 5.8 g/dL. Presented with prostration, tachycardia and polypnea, temperature gradient on leg and pallor of the conjunctiva, palms, and nailbeds, enlarged liver. Blood transfusion given on admission.

Case 2 (late IC_{90}) Received 9.5 mg/kg of rectal artesunate at 0 hr. Screening parasitaemia 11,304/μL, *pf*HRP2 148 ng/mL, Hb 10.1 g/dL. Malnourished (WAZ -2.79; HAZ -2.01). Presented with pain in the epigastric area for suspected gastric ulcer, hematemesis and melena, prostration, compensated shock/temperature gradient on leg, enlarged liver and spleen.

Case 3 (late IC_{90}) Received 8.0 mg/kg of rectal artesunate at 12 hr. Screening parasitaemia 265,770/μL, *pf*HRP2 5,289 ng/mL, Hb 7.8 g/dL. Presented with tachycardia, polypnea and pallor of the conjunctiva, enlarged liver. Blood transfusion given on admission.
